# Case report: Intrapericardial thrombus aspiration in early stage of pericardial thrombosis for cardiac tamponade complicating percutaneous left atrial appendage closure

**DOI:** 10.3389/fcvm.2022.924570

**Published:** 2022-09-08

**Authors:** Bin-Feng Mo, Cheng-Qiang Wu, Qun-Shan Wang, Yi-Gang Li

**Affiliations:** ^1^Department of Cardiology, Xinhua Hospital Affiliated to Shanghai Jiao Tong University School of Medicine, Shanghai, China; ^2^Department of Cardiology, The First Affiliated Hospital of Guangxi University of Chinese Medicine, Nanning, China

**Keywords:** thrombus aspiration, pericardial thrombosis, cardiac tamponade, left atrial appendage closure, case report

## Abstract

**Introduction:**

Pericardial thrombosis that complicates pericardial bleeding is a life-threatening emergency in interventional cardiology, and surgery remains the only definitive treatment option. We report the first case of successful intrapericardial thrombus aspiration using a dedicated thrombus aspiration catheter in the early stage of pericardial thrombosis.

**Case report:**

A 76-year-old woman with non-valve atrial fibrillation underwent percutaneous left atrial appendage (LAA) closure for secondary prevention of stroke. A 24-mm Watchman device was deployed under fluoroscopic guidance. Post-deployment angiography revealed LAA perforation, which led to the rapid onset of cardiac tamponade. Emergency pericardiocentesis was performed and the deep-seated device was redeployed at a more proximal position to seal the distal perforation. Pericardial bleeding was controlled after the drainage of 400 ml of blood. However, the patient re-developed cardiac tamponade following a period of stability. The patient was diagnosed with early-stage pericardial thrombosis based on echocardiographic findings of a hypoechoic effusion in the pericardial space. Thrombus aspiration using a pigtail catheter and long sheath failed; however, we performed successful intrapericardial thrombus aspiration using a dedicated thrombus aspiration catheter. We drained 120 ml of sludge-like blood, and the patient underwent successful conservative management without surgical intervention.

**Conclusion:**

This case report highlights the potential usefulness of a percutaneous intrapericardial thrombus aspiration technique using a dedicated thrombus aspiration catheter in selected patients with early-stage pericardial thrombosis, as a less invasive alternative to cardiac surgery.

## Introduction

Cardiac tamponade is a potentially fatal complication in patients who undergo percutaneous cardiac interventions (1–3). Procedural-related cardiac or coronary perforations can cause rapid accumulation of blood in the pericardium and may precipitate a cardiac emergency (4). Emergency pericardiocentesis is a life-saving procedure in hemodynamically unstable patients with clinical evidence of cardiac tamponade (5).

Reportedly, the incidence of pericardial effusion that necessitates intervention was 4.8 and 1.9% in randomized left atrial appendage closure (LAAC) trials of PROTECT AF and PREVAIL, respectively (6, 7). More recent studies have reported a lower pericardial effusion rate of less than 1–2% (8, 9). Most intervention-induced perforations (approximately 88%) can be treated conservatively using pericardiocentesis, without surgical intervention (5). However, open surgical repair is necessary for patients with prolonged uncontrolled bleeding or pericardial thrombosis (10). The latter is a more fatal complication; pericardial thrombus significantly restricts the diastolic function of the heart (4) and cannot usually be aspirated using a pericardial drainage tube. We report a case of successful intrapericardial thrombus aspiration using a dedicated thrombus aspiration catheter during early-stage pericardial thrombosis, as a less invasive alternative to cardiac surgery.

## Case

A 76-year-old woman with a history of non-valve atrial fibrillation (AF), hypertension, diabetes, prior ischemic stroke, a CHA_2_DS_2_-VASc score of 7, and a HAS-BLED score of 3 underwent percutaneous LAAC for secondary prevention of stroke.

**FIGURE 1:**
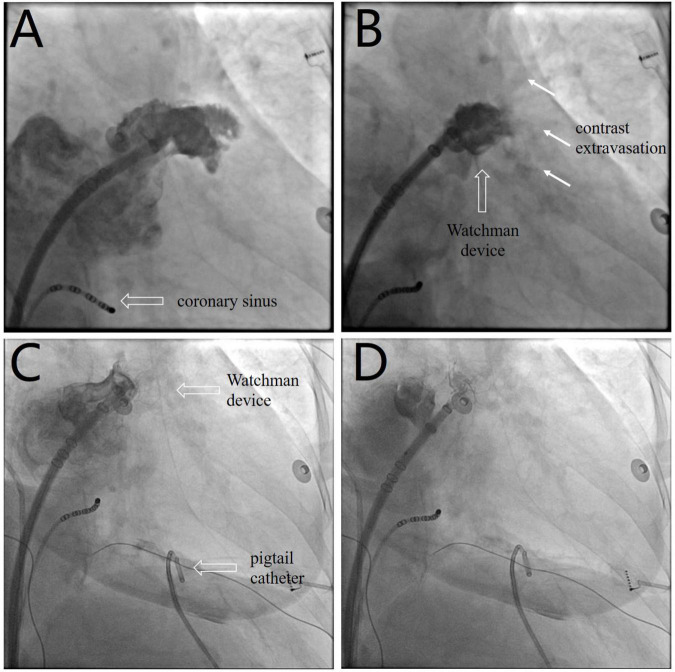
**(A)** Left atrial appendage angiography before device selection (Supplementary Video 1). **(B)** Post-deployment angiography shows brisk contrast extravasation (white arrow) into the pericardial space (Supplementary Video 2). **(C)** The device is redeployed at a more proximal position to seal the appendage and distal perforation (Supplementary Video 3). **(D)** The device is released after confirmation of device stability and the absence of residual peri-device leakage (Supplementary Video 4).

The LAAC procedure was performed under local anesthesia, deep sedation, and fluoroscopic guidance. A decapolar catheter was inserted through the right femoral vein into the coronary sinus to guide transseptal puncture. Unfractionated heparin (100 U/kg) was administered immediately following transseptal puncture to achieve an activated clotting time of 330 s. The left atrial pressure was 33/15 mmHg. Left atrial appendage (LAA) angiography (Figure 1A and Supplementary Video 1) at the right anterior oblique 30° and caudal 20° view revealed ostial width of 19.5 mm and depth of 21.8 mm. A 24-mm Watchman device (Boston Scientific, MA, United States) was selected and then deployed under fluoroscopic guidance. Post-deployment angiography revealed brisk contrast extravasation into the pericardial space (Figure 1B and Supplementary Video 2). The patient rapidly developed cardiac tamponade, and blood pressure decreased from 131/72 to 78/35 mmHg.

We performed emergency pericardiocentesis via a subxiphoid approach under fluoroscopic guidance. A pigtail catheter was inserted into the pericardial cavity to drain blood, and the aspirated pericardial blood was immediately returned to the femoral vein via a sheath. Protamine (30 mg) was simultaneously administered to reverse heparin activity. The patient’s systolic blood pressure returned to 95 mmHg after aspiration of 150 ml of blood. The device was retracted and redeployed at a more proximal position to effectively seal the LAA and distal perforation (Figure 1C and Supplementary Video 3). After confirmation of device stability and absence of residual peri-device leakage, the Watchman device was released (Figure 1D and Supplementary Video 4). Repeat aspiration was performed for an additional 15 min. The patient’s vital signs stabilized, and blood pressure returned to 120/65 mmHg. The pericardial fluid was drained to dryness after aspiration of 400 ml of blood, and minimal pericardial fluid reaccumulation was observed.

**FIGURE 2:**
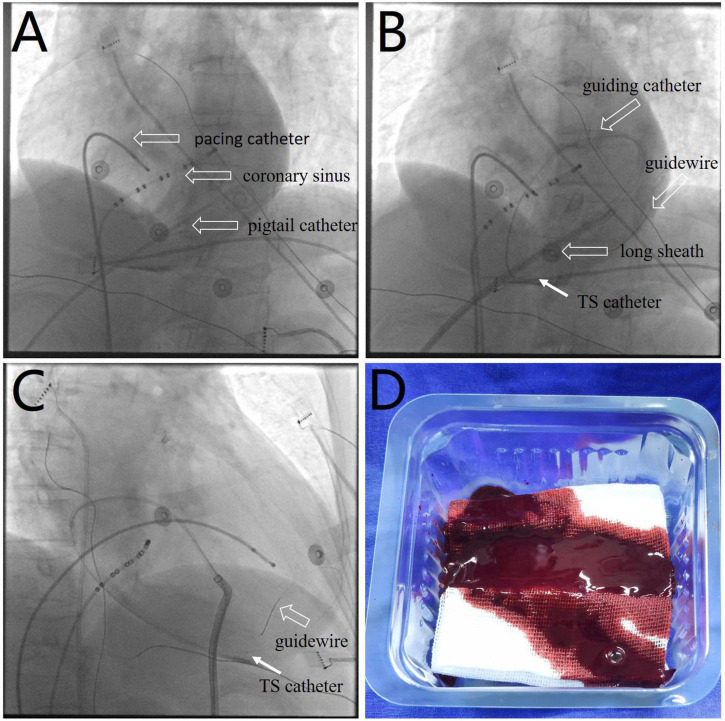
**(A)** Fluoroscopic image showing a near-normal sized cardiac silhouette (Supplementary Video 5), although the patient’s blood pressure decreased to 90/62 mmHg. **(B,C)** Images showing the use of a thrombus aspiration catheter (white arrows) for thrombus aspiration from multiple pericardial sites with cautious manipulation of the guiding catheter and guidewire (Supplementary Videos 6, 7). **(D)** Image showing a sludge-like appearance of blood (as opposed to a formed thrombus) drained by the aspiration catheter. TS, thrombus aspiration.

**FIGURE 3:**
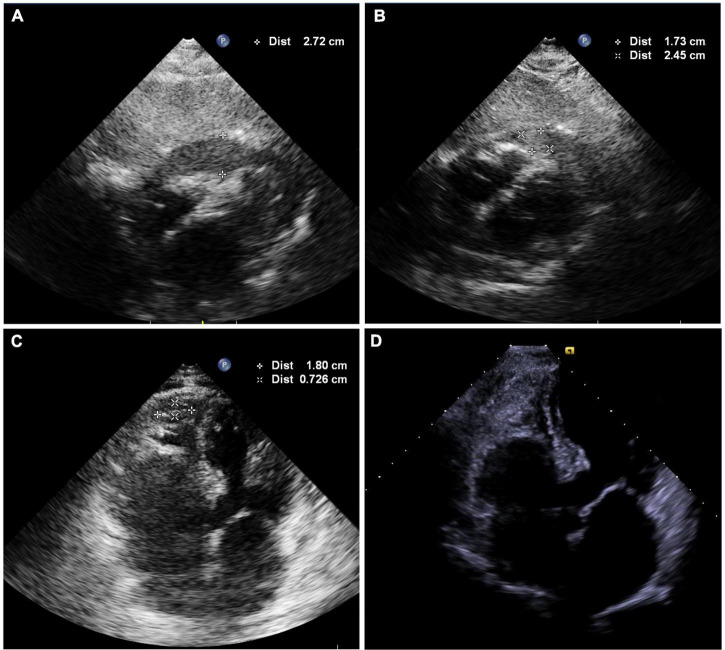
**(A)** Echocardiography showing hypoechoic (rather than anechoic) areas in the pericardial space. **(B)** Echocardiography shows mild effusions with a round hyperechoic thrombus in the vicinity of the right ventricular apex after intrapericardial thrombus aspiration. **(C)** Image showing shrinkage of the round thrombus, which appears as a strip in the vicinity of the right ventricular apex on the second postoperative day (Supplementary Video 8). **(D)** Echocardiography was obtained 2 weeks after discharge and showed no thrombus or pericardial effusion (Supplementary Video 9).

Following 10 min observation, the patient’s heart rate showed intermittent slowing with a decrease in blood pressure to 90/62 mmHg. A temporary pacing lead was placed into the right ventricular to maintain a ventricular rate of > 60 beats per minute. Fluoroscopy revealed a near-normal-sized cardiac silhouette (Figure 2A and Supplementary Video 5), and minimal pericardial blood was drained after the insertion of a new pigtail catheter. The decrease in blood pressure was likely attributable to suspected pericardial thrombosis. Emergency echocardiography revealed a hypoechoic (rather than anechoic) effusion in the pericardial space (Figure 3A), suggestive of early pericardial thrombosis. The emergency surgical team was summoned to prepare for open chest surgery.

The patient’s blood pressure was temporarily stable at approximately 90/60 mmHg; therefore, intrapericardial thrombus aspiration was attempted before surgery. However, thrombus aspiration failed using a pigtail catheter, which was replaced by an 8.5 F long sheath (SL1, Abbott, MN, United States). Unfortunately, thrombus aspiration through the sheath was also unsuccessful. Thereafter, we used a dedicated thrombus aspiration catheter. A 6F guiding catheter (Judkins R4.0, Medtronic, MN, United States) was inserted through the long sheath into the pericardial cavity via an angioplasty guidewire (BMW, 0.036 cm × 190 cm, Abbott, MN, United States), and a thrombus aspiration catheter (Thrombuster II, Kaneka Medical Products, Osaka, Japan) was advanced into the pericardial cavity via the guidewire. Following manipulation of the guiding catheter and guidewire, we could maneuver the thrombus aspiration catheter to successfully aspirate the thrombus from multiple sites across the pericardium (Figures 2B,C and Supplementary Videos 6, 7). Sludge-like blood (instead of a thrombus) was drained using the aspiration catheter (Figure 2D). We aspirated 120 ml of sludge-like blood after 10 min. The patient was hemodynamically stable, and blood pressure returned to 123/62 mmHg. Echocardiography revealed mild effusion and a round hyperechoic thrombus (2.5 cm × 1.7 cm) in the vicinity of the right ventricular apex (Figure 3B). A pigtail catheter was placed to monitor the pericardium, and the patient was transferred back to the ward.

The pigtail catheter was removed on the second postoperative day after echocardiography confirmed the absence of pericardial fluid reaccumulation. We observed shrinkage of the round thrombus, which appeared as a strip that measured 1.8 cm × 0.7 cm in size near the right ventricular apex (Figure 3C and Supplementary Video 8). Anticoagulation was re-initiated on the third postoperative day, and the patient was discharged on the fifth postoperative day. Echocardiography performed 2 weeks after discharge revealed no thrombus or pericardial effusion (Figure 3D and Supplementary Video 9), and the patient had no thromboembolic event or pericardial effusion during 1-year follow-up.

## Discussion

Pericardial thrombosis is a life-threatening emergency, and therapeutic pericardiocentesis is challenging. We report the first case of successful intrapericardial thrombus aspiration using a dedicated thrombus aspiration catheter in the early stage of pericardiac thrombosis complicating LAAC, providing a percutaneous treatment of early pericardial thrombosis.

Percutaneous LAAC serves as an alternative to oral anticoagulation for stroke prevention in patients with non-valve AF by mechanical occlusion of the LAA. The LAA is a thin-walled structure (11); therefore, it is highly vulnerable to tears or perforation following manipulation by a closure device or catheter, which may lead to pericardial bleeding and potential cardiac tamponade. The rate of pericardial tamponade that complicates LAAC has decreased owing to the implementation of standardized operative procedures and greater accumulation of surgical experience (12); however, pericardial tamponade remains a serious complication of percutaneous LAAC.

Emergency pericardiocentesis is performed to relieve cardiac tamponade in patients with hemodynamic instability. Pericardiocentesis is an effective conservative treatment approach in most patients with pericardial tamponade that complicates LAAC (5). In our case, the closure device was deployed too deep and perforated the LAA, which precipitated pericardial tamponade. Emergency pericardiocentesis was performed to relieve cardiac tamponade. The deep deployed device was redeployed at a more proximal position to seal the distal perforation, and heparin was reversed. These measures effectively controlled pericardial bleeding.

However, the patient returned with cardiac tamponade again after a period of stability and was diagnosed with pericardial thrombosis, following echocardiographic evaluation. Surgical treatment is the only definitive therapeutic option available for pericardial thrombosis (13) and to date, no study has reported successful intrapericardial thrombus aspiration in such cases. Temporary stabilization of the patient’s blood pressure facilitated intrapericardial thrombus aspiration under backup surgery. After the failure of the pigtail catheter and long sheath aspiration, intrapericardial thrombus aspiration was successful using the thrombus aspiration catheter (Thrombuster II), which is attributable to the following advantages offered by this novel approach. The Thrombuster II catheter with a large circular lumen and superior aspiration ability is dedicated for thrombus aspiration. Echocardiography revealed hypoechoic and not anechoic or hyperechoic effusion, which suggested early-stage pericardial thrombosis; incomplete thrombus formation enabled aspiration and the sludge-like quality of drained blood confirmed this finding.

Intra-procedural surveillance of transesophageal echocardiography (TEE) was not performed, which serves as a significant limitation of this study. TEE enables timely detection of pericardial effusion and thrombosis, and can outline the process from pericardial effusion to thrombus formation. Our case is quite unique because pericardial thrombosis was detected during the early stage in this patient. Emergency surgery is inevitable in patients with complete pericardial thrombosis or hemodynamic instability. We do not recommend the routine use of intrapericardial thrombus aspiration utilizing a thrombus aspiration catheter for the management of pericardial thrombosis; however, in selected cases, it may serve as a potential percutaneous method that obviates the need for open chest surgery and the ensuing morbidity. Whether the closure device should be deployed to seal the site of leakage for tears located within the LAA remains questionable. Our single-center experience supports this operation; however, few studies have discussed this topic, and future investigations are warranted to validate our findings.

## Conclusion

Pericardial thrombosis is a life-threatening emergency, and pericardiocentesis for management of this complication is often challenging; therefore, surgical treatment remains the only definitive therapeutic strategy. In this report, we highlight the effectiveness of a percutaneous technique of intrapericardial thrombus aspiration using a dedicated thrombus aspiration catheter in selected patients with early pericardial thrombosis.

## Data availability statement

The original contributions presented in this study are included in the article/Supplementary material, further inquiries can be directed to the corresponding authors.

## Ethics statement

The studies involving human participants were reviewed and approved by the Ethics Committee of Xinhua Hospital Affiliated to Shanghai Jiao Tong University School of Medicine. The patients/participants provided their written informed consent to participate in this study.

## Author contributions

B-FM and C-QW wrote the manuscript. Q-SW and Y-GL revised the manuscript and were in charge of the design of this study. All authors approved the manuscript for publication.
